# Empowering stroke survivors beyond inpatient rehabilitation: the STRIDE program

**DOI:** 10.3389/fstro.2023.1281703

**Published:** 2023-11-23

**Authors:** Jessica M. Cassidy, Ryan Fitzgerald, Rachel M. Vaughn, Anna Geib, Maureen Marquie, Anna Claire Trei, Blaise Morrison, Michael D. Lewek, John M. Baratta

**Affiliations:** ^1^Department of Health Sciences, University of North Carolina at Chapel Hill, Chapel Hill, NC, United States; ^2^Department of Physical Medicine and Rehabilitation, University of North Carolina at Chapel Hill, Chapel Hill, NC, United States

**Keywords:** stroke, rehabilitation, education, discharge, management

## Abstract

**Objective:**

The timeframe from hospital discharge to the commencement of outpatient therapies represents a crucial yet often overlooked period in post-stroke recovery. We designed an eight-week post-stroke management program (STRIDE, Stroke Management Training and Inpatient Rehabilitation Discharge Education) targeting individuals discharging from an inpatient rehabilitation facility to home. The primary aims of this pilot study were to determine STRIDE feasibility and participant engagement.

**Methods:**

Participants with first or recurrent stroke were enrolled. Each week, participants monitored and recorded their daily activity, completed a 15-min educational module and quiz, and partook in weekly and biweekly communication with a fellow participant and STRIDE coordinator, respectively. Feasibility was evaluated by successful initiation of STRIDE and enrollment of the target population. We also assessed participant adherence and conducted semi-structured exit interviews.

**Results:**

Of the 99 individuals screened, 20 individuals were enrolled (7 females, 28.6 ± 15.7 days post-stroke). Several participants were unable to begin the program (*n* = 6) or complete the program (*n* = 4). Overall, participants completing at least 1 week of STRIDE (*n* = 14) demonstrated adherence with education module and quiz completion and communication with the STRIDE coordinator. Participant feedback from interviews was largely positive, underscoring the value of STRIDE during early post-stroke recovery.

**Conclusions:**

These findings support the feasibility of an initiated multi-faceted stroke management program. Participant dropout was a limitation and serves as a consideration when designing future iterations of STRIDE. With the long-term goal of promoting autonomy and investment in one's continued recovery beyond the inpatient setting, STRIDE bridges the transition from hospital to home.

## Introduction

Compared to individuals of a similar age without stroke, individuals post-stroke exhibit reduced physical activity and elevated sedentary time (English et al., 2016; Mahendran et al., 2016; Ezeugwu and Manns, 2017), which likely contribute to the development of secondary complications and hospital readmission. A crucial yet often overlooked post-stroke recovery timeframe is the period from hospital discharge to the commencement of outpatient therapies. This window delineates an individual's transition from a structured therapeutic environment managed by a diverse team of healthcare professionals (Langhorne et al., 2020) to an environment characterized by isolation, self-reliance, disorganization, and often a lack of accessibility to fundamental resources (Adeoye et al., 2019). Coincidently, this timeframe also represents a period of enriched neuroplasticity potential, whereby underlying cellular and molecular mechanisms can propel functional recovery efforts (Cassidy and Cramer, 2017). During this sensitive period, physical activity as part of sensorimotor rehabilitation may prove more impactful (Dromerick et al., 2021). Yet, plateaus or decreases in physical activity are frequently observed post-stroke. A longitudinal study examining physical activity change over the first 6 months following stroke using accelerometers reported that, though the time spent in an upright position increased during the first month after stroke relative to initial hospitalization, no further changes in activity (e.g. upright time, number of transfers, etc.) occurred over the next five months (Askim et al., 2013). Others have also found that, despite increases in activity during inpatient rehabilitation, significant declines were observed by 6 months post-stroke following hospital discharge (Tieges et al., 2015). Lastly, during the subacute period (days to weeks post-stroke), individuals typically completed an average of 5,535 steps per day—a value that diminishes to <4,100 steps per day in the chronic phase (>6 months post-stroke) which is substantially less than the 8,338 steps on average taken by adults without stroke (Fini et al., 2017). These collective findings underscore low engagement in physical activity and missed opportunities to instill autonomy and investment in individuals' health following a major medical event.

As factors such as fatigue, mood, cognition, and motivation contribute to sedentary behaviors (Hopman and Verner, 2003; Tieges et al., 2015; Thilarajah et al., 2018), it is not surprising that post-stroke wellness strategies focused solely on exercise do not foster lasting lifestyle change (Prince et al., 2014; Martin et al., 2015). Rather, long-term engagement in physical activity following stroke depends on a combination of psychological and social factors encompassing self-efficacy, physical activity beliefs, and social support (Morris et al., 2012). A multi-faceted wellness approach is therefore warranted.

We devised an eight-week program (**S**troke Management **TR**aining and **I**npatient Rehabilitation **D**ischarge **E**ducation, STRIDE) that focused exclusively on individuals preparing to discharge from an inpatient rehabilitation facility (IRF) to home following stroke. The STRIDE program featured three main components entailing [1] activity monitoring using commercially available activity monitors, [2] social support through partnerships with fellow stroke survivors and STRIDE personnel, and [3] weekly education. Forging connections with fellow stroke survivors provides opportunities to exchange personal testimony and support (Damush et al., 2007), while mitigating feelings of depression and/or isolation. The benefits gained from these enhanced personal connections may compel individuals to re-examine preconceived notions of their disability (Graham et al., 2008). Given the advent of inexpensive wearable technology spurring reductions in sedentary behaviors (Fini et al., 2015), combining this technology with social support may yield a synergistic effect in promoting accountability, motivation, and engagement in one's health and recovery post-stroke. The purpose of this pilot study was to determine the feasibility of STRIDE. We defined *feasibility* as successful initiation of each STRIDE-related component (i.e., activity monitoring, weekly education and quizzes, and social support/communication) and our ability to recruit and enroll our target population from the IRF setting. Successful evaluation of STRIDE component initiation was based on the creation, organization, and delivery of content to participants. Examples of component initiation include the construction of weekly educational models in both online and written formats, in-person activity monitor device training at the IRF, and the establishment of virtual links to access weekly quizzes. Relatedly, our primary goal was to determine participation engagement with STRIDE as demonstrated by their adherence with activity monitoring and documentation, correspondence with a fellow STRIDE participant and STRIDE coordinator, and completion of weekly education and quizzes. A secondary and more exploratory goal of this work was to assess the potential individual-level impact of STRIDE by measuring personal factors such as physical activity, self-efficacy, and quality of life at the beginning and end of STRIDE and also by conducting semi-structured interviews upon STRIDE completion.

## Methods

### Participants

We recruited individuals with a first or recurrent stroke (ischemic or hemorrhagic) that received inpatient rehabilitation services in an IRF setting discharging to home. Screening and recruitment occurred over a period of eight months (August, 2021–May, 2022). Eligible participants needed to demonstrate adequate English proficiency and sufficient cognitive function as indicated by a Montreal Cognitive Assessment score of at least 22 points out of a possible 30 points. The STRIDE coordinator met with the potential participants and, if possible, their family members/caregivers ~2–3 days prior to hospital discharge to discuss the program and answer questions. Though we designed STRIDE with the goal to be a standalone program, where the majority of setup of STRIDE components was executed by STRIDE personnel (minimal setup from participants), we documented whether or not the participant had access to family members or caregivers upon discharge. Access to family and caregivers may impact a participant's willingness to participate and complete STRIDE-related activities. For instance, participants may have relied on others for turning on their computer or accessing their email where weekly links to educational modules and quizzes were sent by the STRIDE coordinator. Additionally, if factors pertaining to the participant's functional capacity and/or environment arose, such as vision and hearing impairments and/or lack of internet access or smartphone technology, we provided STRIDE-related materials and meetings in alternative formats. All participants provided written consent as approved by the Institutional Review Board at the University of North Carolina at Chapel Hill.

The STRIDE coordinator distributed program materials just before IRF discharge that included an activity tracking journal, educational content, and an activity tracker (Fitbit Inspire 2). Participants received training on Fitbit use with the intention of them wearing the device for at least one day in the hospital just before the STRIDE program commenced. Written instructions regarding Fitbit care and maintenance (i.e., charging device, collecting relevant measures, etc.) were also provided and reviewed with participants.

### Procedures

Components of the eight-week STRIDE program are described in detail below. It is important to highlight the role of the STRIDE coordinator whose primary responsibility was to establish a partnership with each participant prior to discharge. The STRIDE coordinator was not a trained clinician but possessed an educational background in psychology and had experience working with clinical populations. The coordinator also received relevant training in stroke recovery, rehabilitation, and research procedures to ensure best practices with the consent process and the administration of assessments.

#### Activity monitoring

Participants were expected to wear their activity tracker during waking hours, including during bathing and/or showering. The activity tracker was worn on their paretic wrist primarily to ensure that they could don and doff the device using their non-paretic extremity. At the end of each day, participants documented their step count, calories burned, and distance traveled in their activity journal. Participants later reviewed their activity journal with the STRIDE coordinator during phone calls and/or virtual meetings and/or mailed completed journals back to the coordinator after the eight-week duration using the provided prepaid return envelope.

#### Social support

The STRIDE coordinator arranged a “participant buddy system” comprised of two individuals discharging from the hospital around a similar timeframe and facilitated subsequent introductions between the individuals. Participants were expected to complete at least two phone calls or virtual meetings with their partner each week depending on their communication preference and complete biweekly check-in virtual meetings or phone calls with the STRIDE coordinator to discuss topics pertaining to activity tracking, educational modules, partner communication, and their overall recovery experience and transition to home.

#### Education

Participants were expected to complete a single 15-min education module each week followed by a 5-question quiz. Content delivery methods included videos that participants streamed via a private YouTube channel and/or written content provided to them at IRF discharge. Module topics included general stroke knowledge, secondary conditions (e.g., cognition, sleep health, bowl/bladder, swallowing, hemiparesis, etc.), caregiver roles, nutrition, psychosocial aspects (e.g., depression, self-confidence, emotional lability, changing roles within the family, etc.), aerobic exercise, community resources and support, and safety.

During their initial and final communication with the STRIDE coordinator, participants completed measurements of self-efficacy (Activities-specific Balance Confidence (ABC) Scale), quality of life (Stroke Specific Quality of Life Scale, SS-QOL), global disability (modified Rankin Scale), physical activity (Physical Activity Scale for Elderly, PASE), and mood (Patient Health Questionnaire 8-Item). As a secondary goal of this work, we examined pre-post changes in ABC Scale, PASE, and SS-QOL scores using Wilcoxon Signed Rank tests. To supplement this information, participants also completed a semi-structured interview with a STRIDE team member trained in qualitative research interviewing at the end of STRIDE.

In addition to assessing feasibility on the basis of successful deployment of the above STRIDE-related components and participant recruitment and enrollment, we evaluated STRIDE participant adherence based on [1] consistent daily monitoring and documentation of their physical activity as evidenced by discussions and review of their activity journal with the STRIDE coordinator and the completion of [2] two phone calls or virtual meetings with their STRIDE partner each week, [3] two phone calls or virtual meetings with the STRIDE coordinator each month, and [4] one weekly educational module and quiz.

## Results

**Figure 1:**
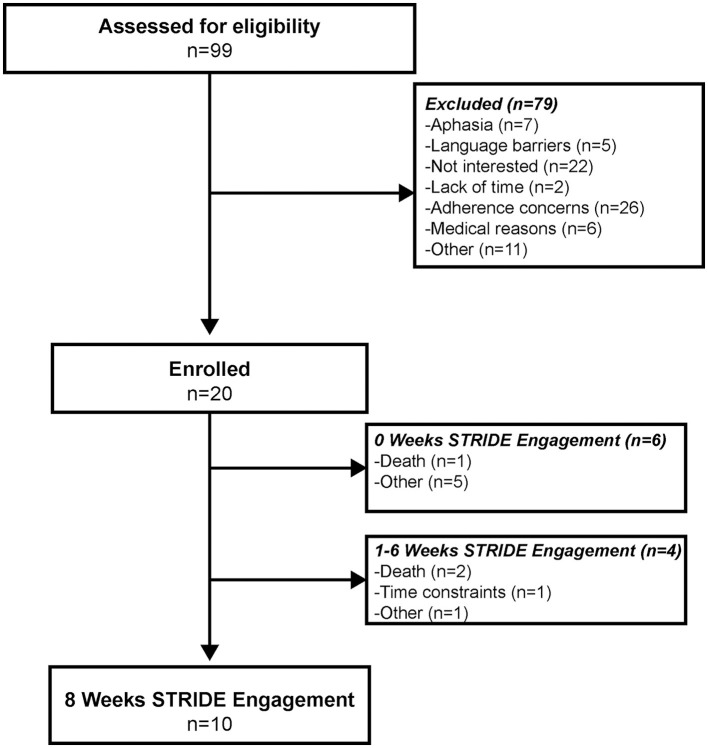
CONSORT diagram illustrating STRIDE screening, enrollment, and participant engagement.

Of the 99 individuals screened, 20 individuals enrolled in STRIDE (Figure 1). Enrollment obstacles included language barriers, lack of interest and/or time, medical reasons beyond immediate stroke, global or receptive aphasia, and concerns of adherence based on participant admission and/or assessed by the STRIDE coordinator and/or physician (JMB). Of the 20 participants enrolled (Table 1), 10 engaged in STRIDE for the full 8 weeks, 4 engaged in STRIDE for at least 1 week, and 6 were unable to begin STRIDE upon IRF discharge. On average, participants began STRIDE approximately 10 ± 5 days (range: 4–23 days) following IRF discharge. Reasons for program attrition or inability to begin STRIDE upon hospital discharge included unanticipated death, time constraints, and other unforeseen obstacles experienced after enrollment.

**Table 1:** Participant demographics and clinical measurements acquired during STRIDE.

**Participant**	**Age (years)**	**Sex**	**Race**	**Prior stroke**	**IRF stay (days)**	**STRIDE weeks completed (max= 8)**	**30–Day hospital ED readmission**	**mRS Pre**	**mRS Post**	**ABC scale pre (%)**	**ABC scale post (%)**	**PASE pre**	**PASE post**	**PHQ−8 pre**	**PHQ−8 post**	**SS–QOL pre**	**SS–QOL post**
ST01	55	M	AA	Y	20	8	N	1	1	62.5	59.7	57.4	76	2	5	131	160
ST02	78	F	C	N	21	6	Y	5	–	1.6	–	82.3	–	11	–	131	–
ST03	60	M	C	Y	26	0	Y	–	–	–	–	–	–	–	–	–	–
ST04	62	M	AA	Y	23	8	N	1	1	30.6	52.5	110.7	175.1	9	5	196	143
ST05	54	M	AA	N	28	0	N	4	–	30.3	–	38.7	–	2	–	209	–
ST06	56	M	AA	N	14	8	N	3	3	52.5	42.8	89.3	64.3	1	2	215	207
ST07	75	F	C	N	9	5	N	2	–	55.6	–	76.4	–	6	–	176	–
ST08	51	M	Biracial	N	17	8	N	3	3	60.6	72.1	46.9	93.65	15	16	141	132
ST09	76	F	C	N	27	1	N	5	–	0.12	–	68.37	–	12	–	90	–
ST10	57	F	AA	N	12	0	Y	–	–	–	–	–	–	–	–	–	–
ST11	46	M	AA	N	42	0	Y	–	–	–	–	–	–	–	–	–	–
ST12	59	M	AA	N	26	8	N	2	2	77.8	79.1	89.6	76.4	2	0	233	180
ST13	54	F	C	N	11	8	N	4	3	35.9	72.2	15.1	9.7	8	9	141	170
ST14	49	F	C	Y	11	0	N	–	–	–	–	–	–	–	–	–	–
ST15	71	M	C	N	21	8	N	3	3	44.1	89.1	76.5	136.8	8	1	172	187
ST16	52	M	C	Y	19	0	N	–	–	–	–	–	–	–	–	–	–
ST17	60	M	C	Y	38	3	Y	1	–	90.3	–	42.9	–	0	–	213	–
ST18	72	M	C	N	14	8	N	–	1	–	79.7	–	154.3	–	2	–	172
ST19	29	M	C	N	21	8	N	3	1	91.3	99.1	33.3	95.16	4	0	211	232
ST20	50	F	AA	N	12	8	N	3	0	61.6	88.4	71.8	65.5	13	14	166	152
Mean ± SD or Median [IQR]	58.3 ± 11.8				20.6 ± 8.9			3 [2–3.75]	1.5 [1–3]	49.6 ± 28.2	73.5 ± 17.5	64.2 ± 26.2	94.7 ± 48.9	7 [2–10.5]	3.5 [1.25–8]	173.2 ± 41.8	173.5 ± 30.0

ED, emergency department; IRF, inpatient rehabilitation facility; mRS, modified Rankin Scale (min-max scores= 0-6 with lower scores indicative of less disability); ABC, Activities-specific Balance Confidence Scale (min-max scores= 0-100% with higher scores indicative of greater balance confidence); PASE, Physical Activity Scale for Elderly (min-max scores= 0-400 or more with higher scores indicative of greater physical activity); PHQ-8, Patient Health Questionnaire 8–Item (min-max scores= 0-24 with higher scores consistent with greater depression symptoms, Scores ≥5, 10, 15, and 20 points consistent with mild, moderate, moderate-severe, and severe depression, respectively); SS-QOL, Stroke Specific Quality of Life Scale (min-max scores= 49-245 with higher scores consistent with greater quality of life); SD, standard deviation; IQR, interquartile range.

### STRIDE feasibility

**Figure 2:**
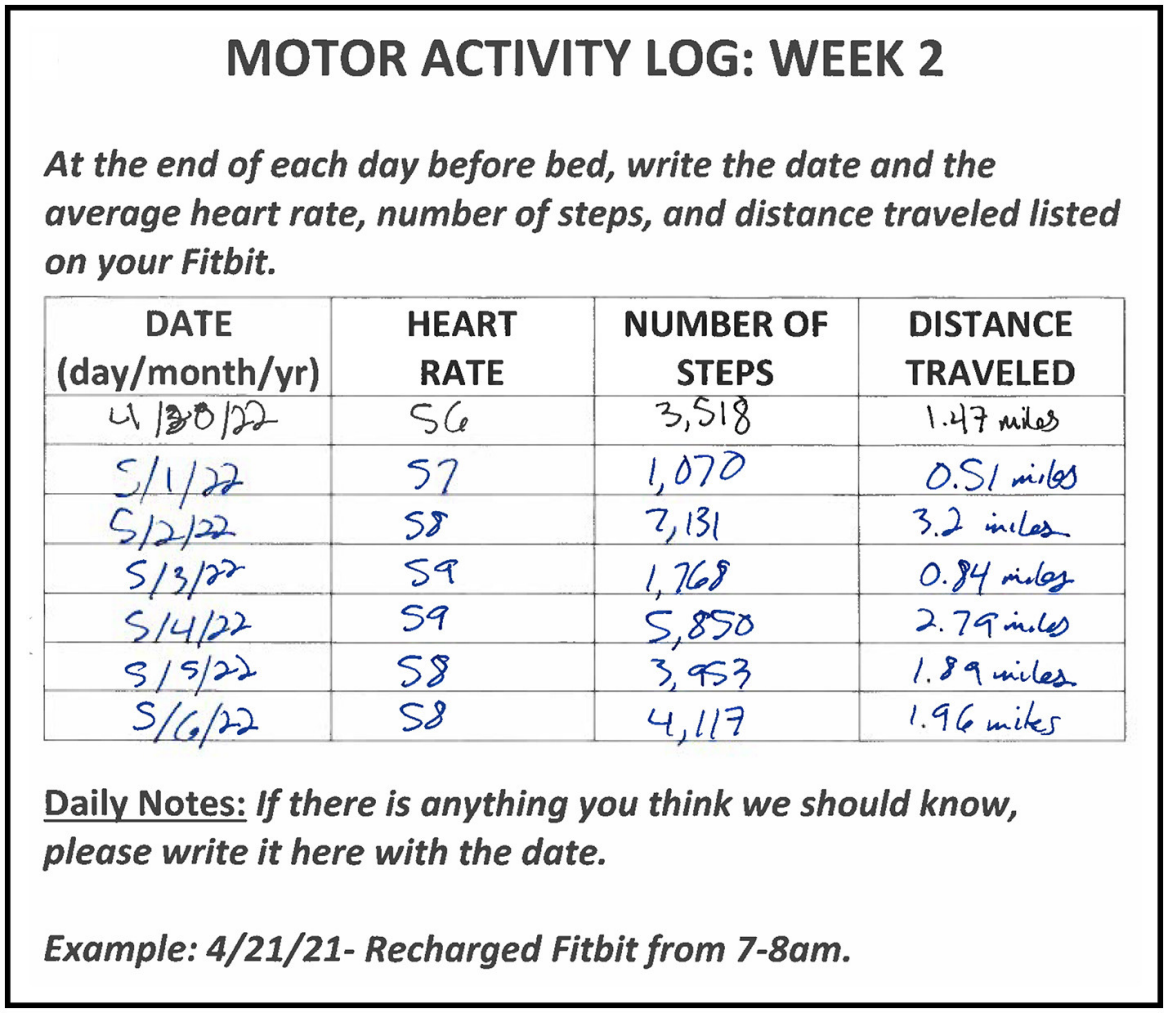
An example of weekly activity monitoring documentation from a participant's motor activity log.

On the basis of successful initiation of STRIDE components (activity monitoring, social support/communication, and completion of weekly education) and recruitment and enrollment of our intended cohort, STRIDE was a feasible program. All but one of the participants owned a Smartphone which enabled STRIDE personnel to create a Fitbit account and connect the Fitbit device to their phone. All participants were successful in learning to use and maintain the Fitbit device as assessed by demonstration to the STRIDE coordinator prior to IRF discharge. For the one participant that did not own a Smartphone, the STRIDE coordinator provided them with a non-Bluetooth pedometer. This individual received similar instruction and encouragement to monitor and record their activity. All participants had internet available in their home to access the STRIDE Youtube channel and view educational modules. Two participants chose to utilize the written vs. online version of the modules. Table 2 summarizes participant adherence to several STRIDE activities. Overall, participants that engaged in all eight weeks of STRIDE demonstrated adherence with Fitbit wear and activity documentation based on activity journal documentation, verbal reports to the STRIDE coordinator during meetings, and/or at exit interviews. Figure 2 provides an exemplar of weekly activity monitoring. Adherence to weekly educational modules and quizzes was also high amongst participants. While initial adherence criteria for communication with the STRIDE coordinator involved bimonthly virtual meetings and/or phone calls, most participants maintained at least a weekly communication schedule with the STRIDE coordinator with encounters ranging from 4 to 70 min in length. Frequent topics of conversation included upcoming medical appointments, educational modules/quizzes, therapy goals, and Fitbit wear. Communication between participants was a less successful component both from an initiation and adherence standpoint. One successful pairing across enrolled participants occurred that resulted in only six phone conversations across the eight-week duration.

**Table 2:** Participant adherence during STRIDE engagement.

**Participant**	**STRIDE weeks completed (max = 8)**	**# of modules completed**	**# of quizzes completed**	**# of phone/virtual meetings with STRIDE coordinator**	**Evidence of activity documentation**
ST01	8	8	8	9	Yes
ST02	6	4	4	6	No
ST03	0	0	0	0	No
ST04	8	8	8	9	Yes
ST05	0	0	0	1	No
ST06	8	8	8	8	No^*^
ST07	5	5	5	5	Yes
ST08	8	8	8	8	Yes
ST09	1	1	1	1	No
ST10	0	0	0	0	No
ST11	0	0	0	1	No
ST12	8	8	8	8	Yes
ST13	8	8	8	7	Yes
ST14	0	0	0	0	No
ST15	8	8	8	8	Yes
ST16	0	0	0	0	No
ST17	3	3	3	5	No
ST18	8	8	8	8	Yes
ST19	8	8	8	10	Yes
ST20	8	8	8	9	Yes

^*^Participant reported using Fitbit device but not documenting activity in journal.

### Individual-level impact

As part of an exploratory assessment, we examined pre/post changes in participant self-reported measures (Table 1). Across the 10 participants that completed the STRIDE program, we did not observe significant changes in self-efficacy (ABC Scale change from 57.4 ± 19.4% to 73.5 ± 17.5%, S= 16.50, *p* = 0.055), physical activity (PASE change from 65.6 ± 30.3 to 94.7 ± 48.8 points, S=11.50, *p* = 0.20), or quality of life (SS-QOL change from 178.4 ± 37.0 points to 173.5 ± 30.0 points, S=-0.50, *p* = 0.99). Participants' feedback was largely positive. Several participants reiterated the importance of peer-to-peer communication with fellow STRIDE participants. Others provided feedback regarding the educational modules. One participant requested additional education about brain anatomy and neurophysiological changes post-stroke. Another participant suggested an alternate ordering of educational modules with the presentation of the “Community Resources” module first so that they could pursue these resources sooner. Table 3 summarizes responses from several participants collected during the semi-structured exit interviews.

**Table 3:** Participant responses from semi-structured exit interviews.

**Describe your experience with the education modules**.
- “I've started uh having some falls, that's new to me, I'm not used to doing any falling. I think it was some of my medication that was changed at the um rehab. But, uhh I reached out to see my primary doctor like [the module] said to I asked [my doctor] and they changed my meds. And so far, it's done pretty good. I'm not as disoriented as I used to be.”
- “It was pretty interesting how some of these um modules really hit the nail on the head, in particular, the one about the caregiver. Yeah, nailed it there… Some of the emotional issues I was having, and unfortunately, I believe my caregivers got the brunt of that, that module talked about that. You know? Well, I guess I go back to the section about communicating with your caretaker. I did do that, there were sometimes where um, she took over, and I did pull her aside and asked her if I could be more of a participant in some of these decisions that are being made… Give me time to be more of a participant.”
- “Basically, the exercise module helped quite a bit because …I have no home care other than my wife and daughter. We don't have any professional home care around this area. And I'm kinda out in the woods.”
**Describe what it was like tracking your daily activity information**.
- “…Do I really have to write all this down? And it was an eye opener. Having to write down the steps and stuff that I took during the day because, you know, for a while I couldn't get up and do stuff on my own, or whatever, and I… it was just an eye opener. It made me realize, OK. I was trying to do what they told me—I'm gonna say my discharge instructions on what I could and could not do—and I realized that I was not getting up and doing as much moving around and walking as I should have been doing. I was doing some, but to me it still wasn't enough. I hadn't gotten back to where I was at before, you know, the stroke and everything.”
**Are there any aspects of the program you plan to continue?**
- “Umm I'll still watch my tracking. I'll still you know, come back and look at it and try to get better about doin' that on a daily thing cause it not only helps me—it helps me in many ways. Um so that's probably the biggest thing that I would take away from it. … “Well I think that by tracking and it… I think tracking is important even though I did poorly. But the tracking you know, lets you have a way to set yourself goals. To have goals to set yourself goals to improve you know walking more or whatever it may be. But continuing to set goals is very important. To me, you know that helps you get better faster and quicker.”
- “Ultimately yes, I probably will be. I'm not one that keeps flip flopping back and forth and change how I do things. So, stuff that I may have started as far as walking more, moving around more, and stuff- stuff like that I will keep doing because that's the way I have to do things now. Ya know? I know my limits, but I have to keep doin'.”
- “I think fortunately, say for me I have friends and family and everything, but yeah, I think for some people, I mean you could, your- your little study here could be a real lifeline them, as it was for me in certain issues. I was feeling a little bit, yeah, stressed out or concerned about something and then [the program coordinator] calls (laughs) and I was able to bore him with my problems. So that's- that's helpful. So, I think your program is very helpful and I hope you keep up with it. And I hope y'all continue.”

## Discussion

This work demonstrates the development and preliminary deployment of an eight-week stroke management program (STRIDE) across individuals recently discharged to home following their IRF stay. The components of STRIDE, encompassing weekly education, communication with peers and the STRIDE coordinator, and activity monitoring, collectively bridged inpatient and outpatient stroke care. The successful execution of most STRIDE-related components in many from our target cohort from the IRF setting support the feasibility of this program. However, as discussed below, several opportunities were also identified for consideration toward future work. Preliminary examination of changes in self-reported measures and semi-structured interviews also signify the potential of individual-level impact.

Considerable challenges arise during the transition from IRF to home, including the continuity of rehabilitation care, feelings of isolation, and confronting and accepting one's disability status (Buntin, 2007; Cott et al., 2007; Duncan et al., 2020). A qualitative study examining the process of community reintegration one-year after stroke reported that one of the most profound challenges for individuals was their adjustment of expectations related to physical participation (Wood et al., 2010). Aligning with a person-centered rehabilitation model (Jesus et al., 2022) that champions autonomy, collaboration, and self-determination, several self-management programs in post-stroke care have emerged (Nott et al., 2021; Caetano et al., 2023). Pivotal elements of these programs typically encompass education, communication, self-monitoring, goal setting, and problem solving (Littlewood et al., 2013; Clark et al., 2020). The STRIDE program incorporated several of these elements in a novel manner.

Weekly education and quizzes enabled participants to gradually build knowledge over time as compared to the presentation of copious amounts of information in a compressed timeframe (i.e., typically during the hospital discharge process). The reinforcement of knowledge in weekly STRIDE educational modules also aligns with effective post-stroke education practices (Cameron, 2013). Participant feedback further highlighted the relevance of educational topics and content provided in STRIDE, with some participants initiating conversations with their physician and/or gaining a greater understanding of how their stroke experience impacts the lives of their family members. In a few cases, participants requested additional educational topics and suggested a different ordering of weekly modules. Based on this feedback, future STRIDE iterations may promote additional autonomy by providing participants with a “menu” of educational modules for them to choose in any order depending on their individual needs.

Another successful component of STRIDE was daily activity monitoring; although, future iterations of STRIDE should adopt more effective strategies for verifying activity documentation in addition to prepaid return envelopes and self-reports to the STRIDE coordinator (Table 2). Verification of activity documentation for future work may therefore entail participants texting photos of their daily or weekly journal entries to the STRIDE coordinator. Apart from this cohort, the general public has quickly adopted the use of fitness tracking technology (e.g., devices and smartphone apps) to monitor physical activity with approximately one in five Americans utilizing a fitness tracking device (Vogels, 2020). As physical inactivity plays a role in various disease processes and economic burden (Ding et al., 2016), the goal of this technology is to facilitate behavioral change (Patel et al., 2015). Work has shown a positive effect on physical activity as measured by an increase in 1,850 steps per day when comparing interventions featuring a fitness tracking technology in comparison to control interventions (Laranjo et al., 2021). Physical activity promotion with fitness tracking technology also extends to clinical populations including stroke (Lynch et al., 2018; Caetano et al., 2023). However, the purpose of the activity monitors in STRIDE extended beyond the promotion of physical activity, as we sought to empower participants through [1] learning new technology, [2] monitoring relevant metrics, and [3] recording these metrics in an activity journal/log. For several participants, the monitors enhanced self-awareness, which sometimes resulted in behavioral modification and goal setting (Table 3). Because our outcome measure in STRIDE focused on adherence rather than a particular activity metric, we were able to broaden our enrollment criteria, thereby, ensuring the accessibility of STRIDE across a wide range of physical ability levels, technology proficiencies, and socioeconomic realities. It is important to acknowledge the increasing utilization of commercially available activity monitors to assess post-stroke mobility (Peters et al., 2021) and the ongoing work needed in this field to determine the accuracy of these devices in stroke (Holubová et al., 2022). A trade-off in our work, which encouraged Fitbit wear on the paretic upper extremity for purposes of donning/doffing ease, was likely data accuracy. Recent work has shown that the accuracy of these devices in stroke varies according to body placement which, in turn, varies according to the level of assistive device use during ambulation (Holubová et al., 2022). Future iterations of STRIDE that may utilize tracker metrics as primary or secondary outcome measures, may therefore revisit tracker placement in order to achieve both donning/doffing ease and data accuracy.

Communication was another critical self-management element implemented in STRIDE, occurring through buddy partnerships and STRIDE coordinator correspondence. Participants adhered to the bimonthly communication schedule with the STRIDE coordinator. In fact, the majority of participants increased their communication frequency with the coordinator to a weekly basis, which underscores the valuable role of the STRIDE coordinator in this program. Self-management programs often feature a “transition coach” that “functions as a facilitator of interdisciplinary collaboration encouraging self-management, modeling empowerment, providing information, and facilitating interdisciplinary collaboration across transitions” (Cott et al., 2007). Our STRIDE coordinator fulfilled several of these functions by establishing partnerships with participants during their IRF stay, initiating communication with participants immediately upon discharge to home, addressing participants' questions based on needs of that individual, and also serving as an active listener for participants. Communication between participants, however, did not flourish. Peer support is a vital component of stroke self-management programs as it cultivates the sharing of testimony and learning amongst individuals (Clark et al., 2020). Our intention was to assemble buddy partnerships between participants with similar IRF discharge timeframes, but this proved challenging due in part to recruiting and enrolling participants sharing similar discharge timeframes. We surmise that a lack of structure or framework embedded in the partnerships was ultimately the reason why this component of STRIDE was not successful. A future approach may entail a second STRIDE coordinator responsible for facilitating partnerships by providing appropriate conversational topics and questions for the participants relevant to the stage of post-stroke recovery while also ensuring that both partners contribute to the conversation. This strategy aligns with group self-management programs that typically rely on a leader or a professional familiar in stroke rehabilitation and recovery (Fryer et al., 2016). Lastly, it is critical to acknowledge that there is no guarantee of successful partnerships formed solely on the basis of stroke as a shared experience. Past qualitative work found that individuals participating in post-stroke peer support groups valued peer interactions to a greater extent when there were contextual similarities amongst peers beyond the common experience of stroke (Morris and Morris, 2012). Future strategies to enhance partnerships may therefore focus more on participants' life experiences and circumstances than on the timeframe of IRF discharge.

This work was not formally powered to identify changes in self-reported outcome measures following STRIDE participation. However, a tendency toward balance self-efficacy gains is encouraging. Nott et al. (2021) reported that self-efficacy mediated the impact of a 12-week self-management program on occupational performance and satisfaction in participants post-stroke. Others have shown balance self-efficacy as a predictor of post-stroke community reintegration satisfaction (Pang et al., 2007), which, collectively, underscores the role of self-efficacy in stroke (Gangwani et al., 2022). We also observed that many participants appeared to increase their self-reported physical activity, and future work may examine relationships between activity monitoring adherence, specific activity variables, and self-report scores (i.e., PASE). Given that STRIDE was only an eight-week program occurring early post-stroke and that community reintegration extends well-beyond this timeframe (Wood et al., 2010), we did not expect substantial changes in self-reported quality of life. As stroke self-management programs have the potential to improve quality of life (Fryer et al., 2016), extending the length of STRIDE to provide support during the first 12–24 months post-stroke may be a beneficial next step. A formal randomized controlled clinical trial is ultimately necessary to determine if changes in self-efficacy, physical activity, and quality of life are due to STRIDE.

To conduct the next iteration of STRIDE using a more formal, controlled study design, it is necessary to acknowledge and critically reflect on the 50% completion rate across enrolled participants. This completion rate represents participant-specific issues arising after STRIDE enrollment and IRF discharge that were beyond the control of STRIDE personnel. Future work will therefore require revision of our enrollment criteria and/or implementation strategy by considering factors related to a potential participant's functional capacity, socioeconomic factors, comorbidities, and discharge environment that may contribute to STRIDE engagement for the full eight-week duration.

### Study limitations

This pilot work contains a few limitations including a small cohort along with program accessibility and inclusivity. We observed an overall reduction of stroke admissions to the IRF during STRIDE recruitment which we attribute to pandemic-related nursing staffing shortages and increased insurance approval barriers. The lack of ethnic diversity and absence of participation from those with moderate to severe aphasia present several opportunities for improvement and enhancement of generalizability: translating educational materials to other languages, utilizing bilingual research staff, and collaborating with speech-language pathologists to implement alternative communication routes with the STRIDE coordinator and buddy (e.g., texting, messaging, etc.) to replace or rely less on verbal information.

A second iteration of STRIDE should also examine potential systems-level change by examining 30-day hospital readmission rates (Ottenbacher et al., 2014) that the Center for Medicare & Medicaid Services now consider a national quality indicator. Daras et al. (2021) recently reported an 11.6% readmission rate amongst Medicare patients receiving inpatient rehabilitation post-stroke, noting that rates varied according to initial motor function, level of dependence, and stroke type (Daras et al., 2021). Future work should therefore also consider the extent to which factors related to participants' initial status and stroke influence STRIDE adherence and completion.

## Conclusions

This preliminary work overall demonstrated the development and successful deployment of an eight-week post-stroke program consisting of weekly education, activity monitoring, and communication during a critical and overlooked timeframe of stroke recovery. Overall findings from this work support the utilization of post-discharge education and coordination, which should remain a key component of future programs.

## Data availability statement

The raw data supporting the conclusions of this article will be made available by the authors, without undue reservation.

## Ethics statement

The studies involving humans were approved by University of North Carolina at Chapel Hill Institutional Review Board. The studies were conducted in accordance with the local legislation and institutional requirements. The participants provided their written informed consent to participate in this study. Written informed consent was obtained from the individual(s) for the publication of any potentially identifiable images or data included in this article.

## Author contributions

JC: Conceptualization, Formal analysis, Funding acquisition, Investigation, Resources, Supervision, Writing—original draft. RF: Data curation, Project administration, Writing—review & editing. RV: Data curation, Resources, Writing—review & editing. AG: Data curation, Resources, Writing—review & editing. MM: Data curation, Writing—review & editing. AT: Data curation, Writing—review & editing. BM: Writing—review & editing. ML: Conceptualization, Writing—review & editing. JB: Conceptualization, Funding acquisition, Writing—review & editing.
